# IR Spectroscopic Characterization of Methane Adsorption on Copper Clusters Cu_*n*_^+^ (*n* = 2–4)

**DOI:** 10.1021/jasms.2c00046

**Published:** 2022-04-12

**Authors:** Olga V. Lushchikova, Stijn Reijmer, P. B. Armentrout, Joost M. Bakker

**Affiliations:** § Institute for Molecules and Materials, FELIX Laboratory, Radboud University, Toernooiveld 7, 6525 ED Nijmegen, The Netherlands; † Department of Chemistry, University of Utah, 315 South 1400 East, Room 2020, Salt Lake City, Utah 84112, United States; + Institut für Ionenphysik und Angewandte Physik, Universität Innsbruck, Technikerstraße 25, 6020 Innsbruck, Austria

## Abstract

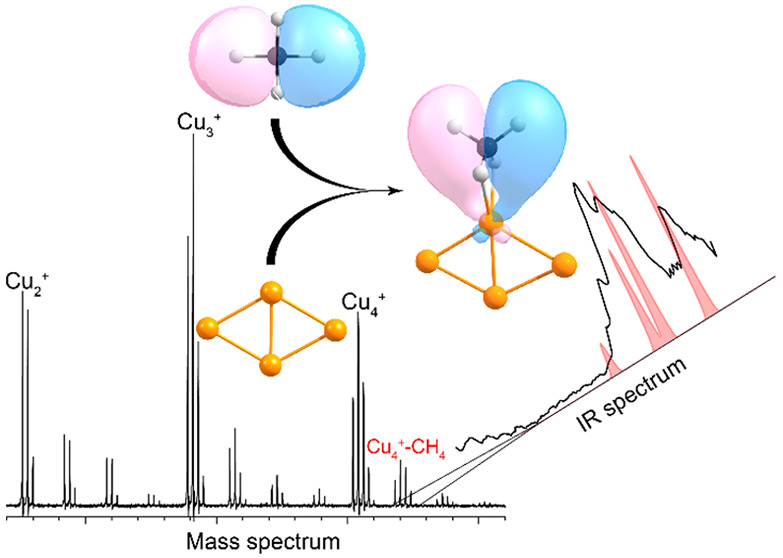

The interaction of CH_4_ with cationic copper clusters has been studied with infrared-multiple photon dissociation (IRMPD) spectroscopy. Cu_*n*_^+^ (*n* = 2–4) formed by laser ablation were reacted with CH_4_. The formed complexes were irradiated with the IR light of the free-electron laser for intracavity experiments (FELICE), and the fragments were mass-analyzed with a reflectron time-of-flight mass spectrometer. The structures of the Cu_*n*_^+^–CH_4_ complexes are assigned on the basis of comparison between the resulting IRMPD spectra to spectra of different isomers calculated with density functional theory (DFT). For all sizes *n*, the structure found is one with molecularly adsorbed CH_4_. Only slight deformations of the CH_4_ molecule have been identified upon adsorption on the clusters, which results in redshifts of the spectroscopic bands. This deformation can be explained by charge transfer from the cluster to the adsorbed methane molecule.

## 1 Introduction

Methane is an abundant component in natural gas and plays an important role as a feedstock for higher-value chemicals, such as olefins or liquid fuels. The chemical utilization of methane currently requires drastic reaction conditions, including the use of aggressive reactants and high temperatures to overcome the thermodynamically very strong C–H bond with its bond energy of 4.56 eV.^1^ It is thus energetically costly to transform methane into syngas (a mixture of CO, CO_2_, and H_2_) via the steam reforming process before using that to form more valuable chemicals, such as larger alkanes through Fischer–Tropsch synthesis.^1^ To reduce the energy cost, it would be advantageous to find catalyst materials that allow bypassing this two-step process and directly functionalize methane. To design such catalysts, it is of interest to find materials that can activate a single C–H bond, leaving the remaining methyl group intact. Such a process would allow for the functionalization of a substrate thermodynamically less stable than methane.

To get a detailed understanding of the metal-methane interaction in possible catalyst materials, reactions between metal ions and clusters are frequently studied in the gas phase. By isolating the reactants and studying the products formed with mass-spectrometric or spectroscopic techniques, it is possible to reconstruct element-, size-, and structure-specific reactivity information with the aid of computational methods. Reactions between metal ions and clusters with methane have been studied extensively.^2−4^ Because of their electrophilic nature, positively charged ions are generally more reactive toward CH_4_ than neutrals or anions.^5^ Products formed when reacting atomic metal ions M^+^ and methane have been widely studied using optical spectroscopy to uncover their structures.^6−23^ Possible binding motifs cover the spectrum from η^3^ for electrostatic dominated interactions and η^2^ for orbital interactions, to C–H activation and subsequent H_2_ elimination yielding a [M,C,2H]^+^ product that can adopt carbene or hydrido carbyne structures. Experimental structural information on reaction products involving cationic clusters is more limited. Iron clusters were shown to only weakly bind methane,^24,25^ whereas rhodium and tantalum clusters activate and dehydrogenate methane.^26,27^ Platinum clusters also dehydrogenate methane, although IR spectra of only an intermediate with elongated C–H bonds were reported.^28^ Recently, it was found that small cationic gold clusters, Au_*n*_^+^ (*n* = 2–4), dominantly bind methane by an η^2^ motif, but indications were found they can also activate a single C–H bond leading to the formation of a hydrido-methyl complex.^29^ A successful catalyst material should be able to activate methane, without suffering from coke formation, as is the case for the highly active Pt.^30^ It has been shown that noble metals like copper, silver, and gold can inhibit coke formation.^31,32^ In light of the recent experimental findings on methane activation by small gold clusters,^29^ it would thus be interesting to find whether silver and copper clusters could also activate methane. Theoretically, it was predicted that both cationic and neutral Cu_20_ clusters are more active than Ag_20_ clusters for methane activation.^33^

We have recently reported IR multiple photon dissociation (IRMPD) spectra of Cu_*n*_^+^·Ar (*n* = 3–10) and of the products resulting from the individual reactions of H_2_/D_2_ and CO_2_, and their combination with Cu_*n*_^+^.^34−36^ Here, we extend our work to reaction products with methane. Because Cu^+^ binds CH_4_ in η^2^ coordination more strongly than Au^+^,^22^ it could be expected that Cu_*n*_^+^ clusters may be more reactive toward CH_4_ than Au_*n*_^+^ clusters. Copper is also known to play a role in natural methane oxidation at ambient conditions, via its presence in the particulate methane monooxygenase (pMMO) enzyme found in the bacteria that use methane as a primary energy source.^37^ This enzyme contains mono-, di-, and trinuclear copper centers, which are involved in the catalytic process, although it is under debate which of these is active in the catalytic conversion of CH_4_ into methanol.^38,39^

The interaction of Cu^+^ with methane was recently studied by Metz and co-workers, who investigated Cu^+^–(CH_4_)_*m*_ (*m* = 1–6) using IR photofragmentation spectroscopy.^22^ They found spectra consistent with intact adsorption for all *m*, with a small red shift of frequencies of the C–H stretching vibrational bands, indicative of a relatively weak interaction. In this work, we employ IR photofragmentation spectroscopy to investigate the reaction products of methane with small cationic copper clusters Cu_*n*_^+^–CH_4_ (*n* = 2–4) in the 200–1650 cm^–1^ spectral range. The structures of Cu_*n*_^+^–CH_4_ complexes are subsequently determined by a comparison of the IRMPD spectra with spectra calculated for different isomers using density functional theory (DFT). We also complement these IR spectra with transition state calculations for hydrido-methyl formation over the bare cluster having structures that are now established.^34^

## 2 Methods

### 2.1 Experimental Section

Copper clusters were produced in a Smalley-type laser ablation source, where a rotating and translating Cu rod was ablated by a pulsed Nd:YAG laser (532 nm) with ∼30 mJ pulse energy in the presence of a He gas pulse, which cools the created plasma and promotes cluster formation in a 3 mm diameter, 60 mm long growth channel.^40^ Methane was injected by a second pulsed valve into an extension of the growth channel (3 mm diameter, 44.5 mm long), where it reacted with the formed Cu clusters. Upon exiting the extended growth channel, the cluster-gas mixture expanded into vacuum through a converging-diverging nozzle with a diameter of ∼0.7 mm. The formed molecular beam was collimated by a 2 mm skimmer and by a 0.45 mm slit, to ensure that all complexes were irradiated by the IR laser beam from FELICE, the free-electron laser for intracavity experiments,^41^ which crossed the molecular beam at a 35° angle. After irradiation, all positively charged species were extracted by pulsed high-voltage plates into a time-of-flight mass spectrometer (TOF-MS, R.M. Jordan TOF Products, Inc.), where they were mass separated and detected by a multichannel plate (MCP) detector. The experiment ran at double the frequency (20 Hz) of the IR laser (10 Hz), allowing alternating mass spectra to be recorded with and without irradiation by IR light. A typical mass spectrum without IR irradiation can be found in the Supporting Information.

The IR radiation employed in this study is in the 200–1650 cm^–1^ range, with macropulse energies ranging between 0.2 and 1.3 J. The spectral bandwidth was set to approximately 0.7% full-width at half-maximum (fwhm) of the central frequency. The fluence of the IR laser can be varied by moving the experimental apparatus along the laser beam in or out of the focus position. The spectra were recorded with the instrument positioned at 115 mm (referred to as “high fluence”) and 280 mm (“low fluence”) from the laser focus. These two positions resulted in fluences ranging from 2 to 10 J/cm^2^ at 280 mm from the focus and 4–45 J/cm^2^ at 115 mm from the focus, with the peak fluence at 1100 cm^–1^.

IRMPD spectra were obtained by monitoring the intensity of the selected mass channel as a function of the laser frequency. Upon resonant IR excitation of vibrational modes of a specific complex, it can fragment leading to a decrease in intensity of the corresponding mass channel. Typically, spectra can be obtained by monitoring the depletion of the specific mass channel, but the presence of mass peaks for Cu_*n*_^+^–(CH_4_)_*m*_, with *m* > 1, can lead to the fragmentation of these species and *growth* of the Cu_*n*_^+^–(CH_4_) channel. Because the focus of this Research Article lies on the complexes with a single CH_4_ molecule adsorbed onto the Cu_*n*_^+^ clusters, we constructed the spectra for Cu_*n*_^+^–(CH_4_) in the following way. First, the branching ratio *B* of the intensities (I) of Cu_*n*_^+^(CH_4_)_*m*_ (*m* = 1–3) clusters to those of all Cu_*n*_^+^(CH_4_)_*m*_ (*m* = 0–3) species was calculated using eq 1.
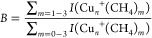
1The fragmentation yield *Y*(ν) at frequency ν was then calculated by taking the natural logarithm of the ratio of the branching ratios with IR irradiation *B*(ν) to that without *B*_0_ using eq 2
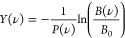
2and normalizing this to the laser macropulse energy *P*(ν). This expression corrects for fragmentation of complexes with multiple CH_4_ adsorbed into the Cu_*n*_^+^–CH_4_ mass channel as well as for fluctuations in the cluster production.

### 2.2 Computational Details

Geometries of Cu_*n*_^+^–CH_4_ were optimized using the Gaussian 16 package^42^ with the Perdew–Burke–Ernzerhof (PBE)^43^ functional with a triple-ζ basis set with two polarization functions (Def2-TZVPP).^44,45^ This functional was chosen because it has successfully explained our previous work on copper cluster cations interacting with Ar, H_2_, and CO_2_.^34−36^ A test calculation on the Cu_2_^+^–CH_4_ system showed that PBE and B3LYP yield very similar predicted IR spectra. Trial geometries of the Cu_*n*_^+^–CH_4_ complexes were constructed by taking the earlier determined structures of cationic Cu clusters^34^ and complexing them with CH_4_ at several adsorption sites and configurations of CH_4_, including structures where CH_4_ was dehydrogenated to form hydrido-methyl complexes. Metal–carbene structures were also considered, however, according to the calculations they are higher in energy (see Supporting Information) and, therefore, unlikely to be formed. Harmonic frequencies were calculated without any symmetry constraints to ensure that proper minima are found and for comparison with the experimental IR spectra. The calculated stick spectra were convoluted with a Gaussian line shape function with a 40 cm^–1^ fwhm. Frequencies are presented unscaled. To evaluate the reaction pathway for potential H migration, transition states were calculated using the same functional and basis set and were verified by frequency calculations to correspond to a true first-order transition state. Once located, such transitions states were verified to connect the desired species by employing the intrinsic reaction coordinate (IRC) method.

## 3 Results

### 3.1 Spectroscopy of Cu_2_^+^–CH_4_

**Figure 1:**
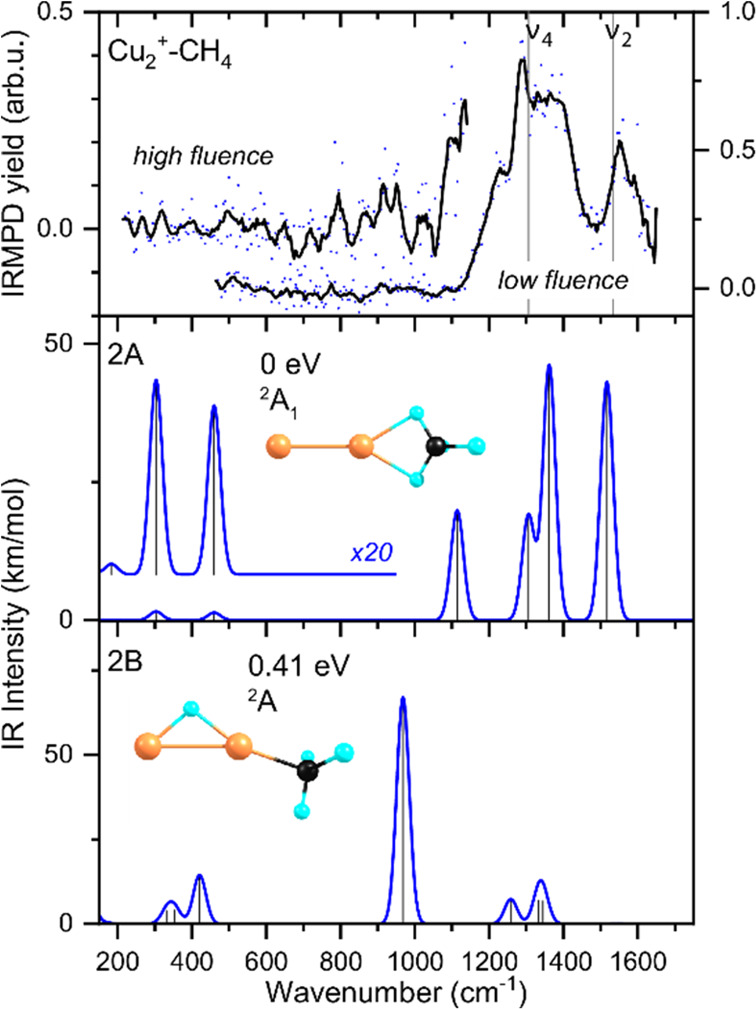
Top panel: Experimental IR spectra of the Cu_2_^+^–CH_4_ products formed upon reacting methane with Cu_2_^+^ clusters, measured at 115 mm (high fluence) and 280 mm (low fluence) from the laser focus. The raw experimental data (blue scatter points) are accompanied by a three-point adjacent average (black line). The gray vertical lines indicate the vibrational frequencies of free CH_4_. Middle and bottom panels (blue trace) show calculated IR spectra of two possible product structures with molecularly (2A) and dissociatively (2B) bound CH_4_. Each structure is accompanied by the energy with respect to the putative global minimum structure and its electronic symmetry. Cu, C, and H atoms are represented by orange, black, and cyan spheres, respectively.

We have obtained IRMPD spectra of the products formed upon reacting methane with Cu_*n*_^+^ (*n* = 2–4). Although we do not know their structures a priori, we adopt the nomenclature Cu_*n*_^+^–CH_4_ for the species under investigation throughout this work. The experimental spectrum of **Cu**_**2**_^**+**^**–CH**_**4**_ is shown in the top panel of Figure 1, recorded under low fluence conditions over the 450–1650 cm^–1^ range (curve labeled “low fluence”). It is dominated by an intense, structured absorption between 1100 and 1450 cm^–1^ and a separate band at 1550 cm^–1^. A closer look at the broader band allows identification of two maxima at 1289 and 1368 cm^–1^ and a low-frequency shoulder at 1228 cm^–1^. No bands were detected below 1000 cm^–1^. To verify the absence of further bands, the spectrum was also recorded with the instrument closer to the FELICE focus, that is, at fluences that are approximately five times higher, now also covering the 200–450 cm^–1^ range. No clear extra bands are detected here, as can be seen in Figure 1. Potential bands in the region between 750 and 1000 cm^–1^ could be discerned, but these are deemed ambiguous because of the low signal-to-noise ratio in this region. Above 1000 cm^–1^, the signal increases, indicating the beginning of a strong band. We assume that this incipient band has the same origin as the broad absorption band in the spectrum recorded under low fluence conditions and that this band is broadened as a result of the higher IR fluence.

To interpret the spectrum, two vertical dashed lines are added to Figure 1a at 1306 and 1534 cm^–1^, indicating the frequencies of the triply degenerate ν_4_ and the doubly degenerate ν_2_ fundamental modes of free methane.^46^ These modes are all associated with methane deformations, where ν_2_ is IR inactive and ν_4_ is strongly IR active. The closeness of the two main bands observed for Cu_2_^+^–CH_4_ to these frequencies suggests that the product observed is a simple adduct.

We further compare the observed spectrum to the calculated spectra of the lowest energy structures found for molecularly (labeled 2A) and dissociatively (2B) adsorbed CH_4_ on Cu_2_^+^. The other examined structures can be found in figure S2 in SI. Structure 2A has methane adsorbed in a bidentate η^2^ configuration to one of the Cu atoms and lies 0.83 eV below the separated Cu_2_^+^ + CH_4_ asymptote. Its structure is of *C*_s_, near-*C*_2*v*_, symmetry; optimization of a symmetrized structure resulted in a slight distortion (1.3 cm^–1^) of the Cu–Cu–CH_2_ angle from 180°, associated with a 20 cm^–1^ normal mode. We interpret this as a numerical glitch, and assume a full *C*_2*v*_ structure. The lowest energy structure for dissociatively bound methane has a methyl bound to one of the Cu atoms, whereas the fourth hydrogen is bound to both Cu atoms in a bridging configuration. These calculated spectra are shown in the middle and lower panel of Figure 1, accompanied by their relative energies. Upon complexation of methane with Cu_2_^+^, symmetry breaking lifts the degeneracies of ν_2_ and ν_4_ and makes the ν_2_ modes IR active. This is clearly visible in the spectrum for structure 2A: four distinct bands are readily discernible at 1114, 1306, 1361, and 1517 cm^–1^; only the ν_2_ mode at 1444 cm^–1^, associated with the two CH_2_ groups twisting out-of-phase along the Cu_2_^+^ axis, has very little IR intensity (0.0011 km/mol).

Although multiple bands for structures 2A and 2B overlap with each other, it appears relatively straightforward to recognize the overall shape of the spectrum predicted for the molecular adsorption product 2A in the experimental spectrum: both observed bands at 1550 cm^–1^ and around 1300 cm^–1^ are mirrored in the predicted bands at 1517, 1361, and 1306 cm^–1^. The predicted band at 1114 cm^–1^ is the only band not clearly replicated in the experimental spectrum. This mode is associated with a concerted motion of the bound hydrogen atoms through the Cu–H–C plane, and one could speculate that the 1228 cm^–1^ shoulder is associated with this predicted mode, but that assignment would represent a significant mismatch between theory and experiment. The hydrido-methyl structure, 2B, is energetically less favorable than 2A by 0.41 eV and can be discarded as a candidate for the spectrum observed because no band is observed at 968 cm^–1^, an umbrella-type motion of the three methyl hydrogens, which dominates the predicted spectrum. Therefore, we can unambiguously assign the experimental spectrum to a cluster with molecularly adsorbed CH_4_. A dissonance in the comparison between experimental and calculated spectra is the lack of bands observed below 600 cm^–1^, where two bands with only low intensity (1.6 and 1.3 km/mol) are predicted at 304 and 460 cm^–1^. We can speculate that these bands would have been observed with a higher signal-to-noise ratio of the spectrum. In contrast, we will see for the larger Cu_3_^+^ and Cu_4_^+^ complexes that the analogous predicted band, a rocking motion of the methane in the Cu–Cu–H plane, is tentatively observed, despite its lower predicted intensity of 0.4 km/mol.

### 3.2 Spectroscopy of Cu_3_^+^–CH_4_

**Figure 2:**
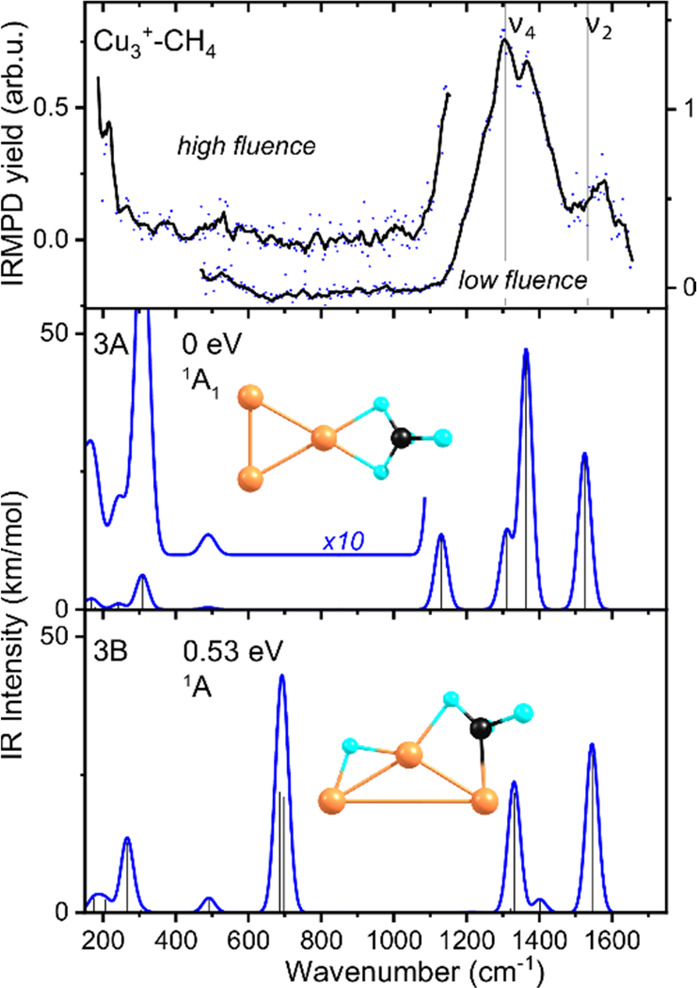
Top panel: Experimental IR spectra of Cu_3_^+^–CH_4_. Middle and bottom panels: Calculated IR spectra of two possible product structures with molecularly (3A) and dissociatively (3B) bound CH_4_. For further details, see caption Figure 1.

The experimental spectrum recorded for **Cu**_**3**_^**+**^**–CH**_**4**_ (Figure 2) is quite similar to that of Cu_2_^+^–CH_4_ in the 800–1650 cm^–1^ spectral range with an intense, structured absorption above 1100 cm^–1^ (submaxima at 1300 and 1368 cm^–1^) and a weaker band peaking just above 1500 cm^–1^. However, in contrast to the spectrum for Cu_2_^+^–CH_4_, there is now also a band at the low-frequency edge of the spectral range under study, at 200 cm^–1^, and potentially features at 375 and 533 cm^–1^, the latter visible both in the low- and high-fluence spectra.

The calculated structures (for more structures see Figure S3) for CH_4_ adsorbed onto Cu_3_^+^ are slightly more separated in energy than for Cu_2_^+^ (structure 3B is 0.53 eV higher in energy than 3A). The binding motif of CH_4_ in the molecularly adsorbed complex 3A (*C*_2*v*_ symmetry) is again η^2^ with a binding energy of 0.75 eV. The spectrum is very similar to that for 2A, signaling the interaction is similar, too. In contrast, the spectrum for dissociatively bound CH_4_ (3B) is significantly different from that for 2B, reflecting the η^2^ binding motif of the methyl group (as opposed to the η^1^ for 2B). Comparison with the experimental spectrum again points at the predominant presence of the molecularly bound isomer 3A. The main factor for rejecting structure 3B is the absence of the intense doublet of bands at 687 and 698 cm^–1^, which are associated with methyl rocking vibrations. The match between 3A and the experiment is again not without fault for the mode predicted at 1130 cm^–1^, the concerted motion of the bound hydrogen atoms through the Cu–H–C plane.

The experimental spectrum also exhibits (the start of) a band at 200 cm^–1^, and it is of interest to see how this compares to calculations. First, we note that no direct match for the strongest band predicted in this range, at 308 cm^–1^, is observed. Because the (start of the) band observed is not readily assignable to any of the other predicted bands, we speculate that it could be due to this 308 cm^–1^ band. It is associated with a stretching motion between the Cu_3_^+^ trimer and CH_4_, for which the exact interaction between the two species is of course very important. One could further tentatively assign the potential feature at 533 cm^–1^ to a weak (0.4 km/mol) predicted band at 489 cm^–1^, a methane rocking mode through the Cu–Cu–Cu plane.

Upon inspection of the lowest-energy dissociative structure, it is of interest to note the significant geometric distortion of the Cu_3_^+^ cluster geometry toward a more linear structure. In structure 3A, the Cu–Cu–Cu angles are still relatively similar to 58° at the apex where CH_4_ is bound, and 61° at the other two corners (compared to the equilateral triangle having 60° angles for the bare Cu_3_^+^). In the dissociative complex, the angles are 118° and twice 31° and the Cu–Cu bond that is not bridged is lengthened to 4.11 Å (from 2.37 Å in Cu_3_^+^), while the others remain relatively unchanged at 2.41 Å each.

### 3.3 Spectroscopy of Cu_4_^+^–CH_4_

**Figure 3:**
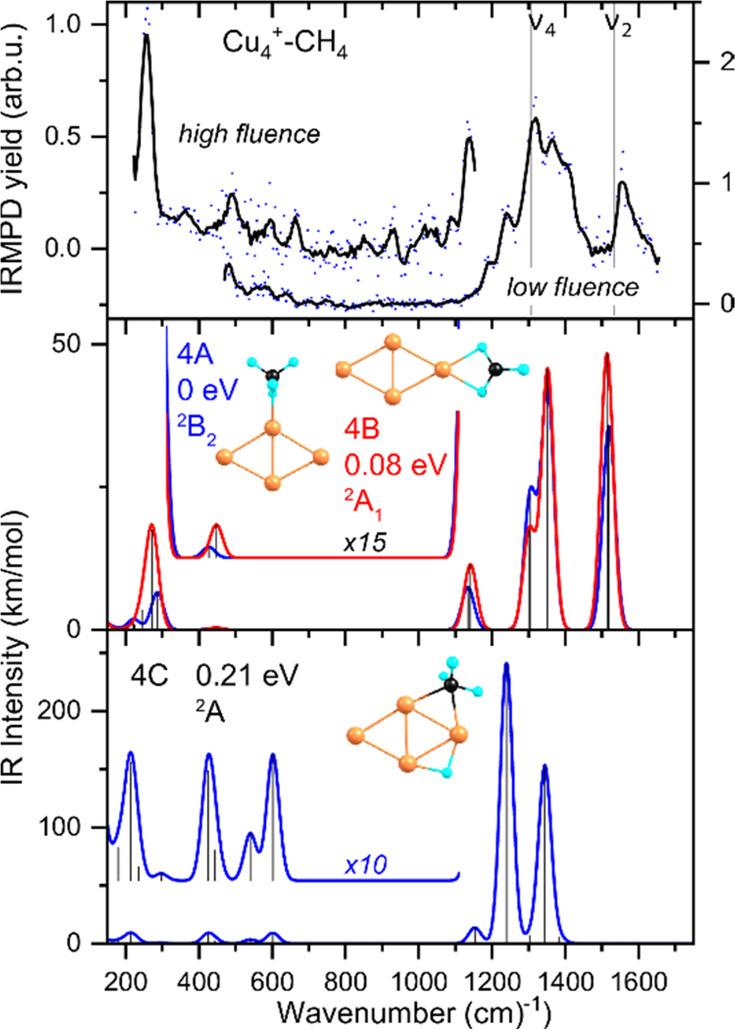
Top panel: Experimental IR spectra of Cu_4_^+^–CH_4_. Middle and bottom panels: Calculated IR spectra of three possible product structures with molecularly (4A, 4B) and dissociatively (4C) bound CH_4_. For further details, see caption Figure 1.

In accord with the spectra for Cu_2_^+^–CH_4_ and Cu_3_^+^–CH_4_, the spectrum of **Cu**_**4**_^**+**^**–CH**_**4**_ (Figure 3) shows a high-frequency region that is composed of several resonances forming a broad, structured band. The overlapping bands are maybe slightly better resolved than those for Cu_2_^+^–CH_4_ and Cu_3_^+^–CH_4_. Here, we identify resonances at 1192, 1243, 1319, 1366, and 1558 cm^–1^, respectively. The spectrum exhibits a pronounced low-frequency band at 256 cm^–1^ and potentially one at 492 cm^–1^.

The experimental spectrum for Cu_4_^+^–CH_4_ is also compared to the calculated spectra of molecularly and dissociatively adsorbed CH_4_ on Cu_4_^+^, Figure 3 (more in Figure S4). Because Cu_4_^+^ is rhombic,^34^ its acute and obtuse apexes offer two inequivalent positions where CH_4_ can adsorb. The resulting structures 4A (adsorption on the obtuse apex, bound by 0.62 eV) and 4B (acute apex) are shown together in the middle panel of Figure 3. These two structures, both of *C*_2*v*_ symmetry, differ by only 0.08 eV, and their spectra are indistinguishable at the current experimental resolution. An isomer with CH_4_ dissociatively bound, structure 4C at +0.21 eV, is shown in the bottom panel.

The diagnostic bands for each of the molecularly bound isomers readily overlap with the observed bands in the high-frequency range. The symmetric scissoring motion is predicted at 1519/1514 cm^–1^ (values for 4A/4B), which corresponds to the experimental band at 1558 cm^–1^. Two bands are predicted at 1305/1302 and 1352/1352 cm^–1^ for the antisymmetric scissoring mode and the Cu–H stretch with simultaneous CH_2_ out-of-plane bending. These bands agree well with the experimental bands at 1319 and 1366 cm^–1^. In the lower frequency range, the unambiguous assignment to 4A/4B is less obvious. Here, the 4A/4B spectrum can explain only two experimental bands at 256 and, potentially, 492 cm^–1^, corresponding to stretches between CH_4_ and the cluster at 287/273 and 427/448 cm^–1^, respectively. However, the bumps observed in the 600 cm^–1^ area could potentially be explained with a 4C spectrum, where two rocking motions of the methyl group at 540 and 602 cm^–1^ are predicted.

### 3.4 Potential Energy Surface for Methane Activation

**Figure 4:**
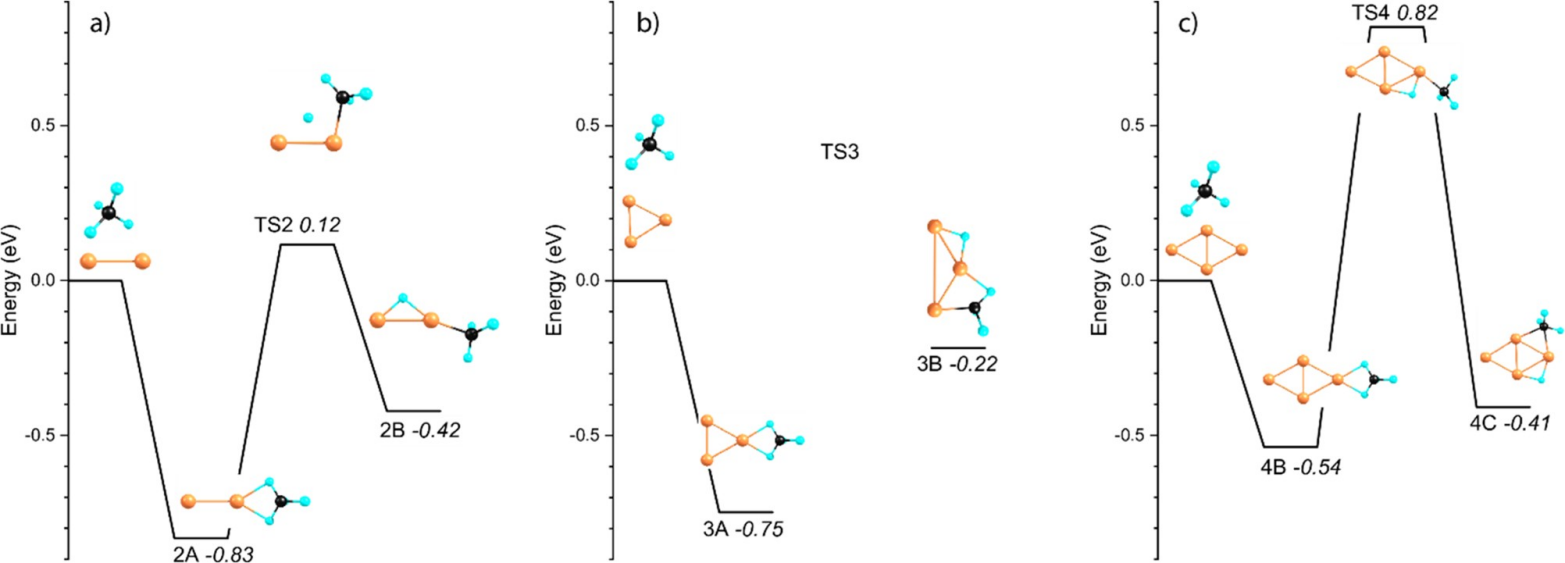
Reactive potential energy surfaces for formation of H–Cu_*n*_^+^–CH_3_ after the adsorption of CH_4_, followed by C–H cleavage and methyl formation. The energy zero corresponds to the separate reactants.

Because we see the clear presence of bands caused by molecularly adsorbed CH_4_ in the experimental spectrum for all cluster sizes studied, their presence in the molecular beam is undebatable. However, the weaker signal around 600 cm^–1^ observed for Cu_4_^+^–CH_4_ could point to the presence of dissociated species formed in the molecular beam. Although 4A and 4C are not very different in energy (0.21 eV), the spectrum is clearly dominated by the entrance complex structures 4A or 4B, and the formation of H–Cu_4_^+^–CH_3_ is likely kinetically hindered. To check this, and to evaluate the trends evolving with cluster size, we have performed transition state calculations linking the molecular adsorption structures with the lowest-energy hydrido-Cu_*n*_^+^-methyl structure found. The reactive potential energy landscapes from adsorption to dissociation are shown in Figure 4, with energies relative to the reactants.

The potential energy surface for methane activation over Cu_2_^+^ linking structures 2A and 2B is shown in Figure 4a. In this process, the η^2^-bound methane migrates away from the symmetry axis, transferring one H to form a Cu–H–Cu structure and then veering back. The associated barrier found is only slightly endothermic (+0.12 eV). The activation reaction for Cu_4_^+^ (Figure 4c) is analogous: the η^2^-bound methane of structure 4B rotates away, transferring H along the rhombus rim to form structure 4C. The barrier found for Cu_4_^+^ is significantly higher than for Cu_2_^+^, 0.82 versus 0.12 eV above reactants (or alternatively, 1.36 versus 0.95 eV above the bottom of the Cu_*x*_^+^–CH_4_ well). For both cluster sizes, the barrier for C–H bond activation, which is 4.5 eV in the absence of the metal cluster, is thus significantly reduced to +0.8 eV (Cu_4_^+^) and even 0.12 eV (Cu_2_^+^). Interestingly, in contrast to the spectrum for Cu_4_^+^, where potential bands that could be indicative for methane activation, were observed, for Cu_2_^+^ no such indications were found, despite the lower barrier. One possible explanation for this is the higher stability of molecularly bound CH_4_ in comparison to the H–Cu_2_^+^–CH_3_ structure (−0.83 vs −0.42 eV or a difference of 0.41 eV) in comparison with Cu_4_^+^ (−0.54 vs −0.41 or a difference of only 0.13 eV). However, the barrier for activation over Cu_4_^+^ is sufficiently high that the dissociative adsorption of CH_4_ onto Cu_4_^+^ must be kinetically hindered. Strangely, no analogous transition state for Cu_3_^+^ could be found, in part because the Cu_3_^+^ cluster deforms considerably upon dissociative adsorption (in contrast to Cu_2_^+^ and Cu_4_^+^). All attempts at stabilizing a structure while breaking the C–H bond and elongating the Cu–Cu bond in the direction of structure 3B failed.

One may then ask whether binding to Cu_*n*_^+^ affects the methane at all (and vice versa). The DFT calculations indicate that CH_4_ undergoes a similar deformation upon adsorption onto all three cluster sizes. The hydrogens bound to the cluster (H_b_) and carbon form the same HCH angle as the two unbound hydrogens (H_u_) (approximately 119°), which is ∼10° larger than that of a free CH_4_ molecule. Simultaneously, the H_b_CH_u_ angle becomes ∼106°, which is 3° smaller than free CH_4_. The C–H_b_ bond length increases by ∼0.03 Å, while free C–H_u_ bonds are shortened by only 0.002 Å, Table 1. This means that the CH_4_ molecule gets wider in the planes parallel and perpendicular to the cluster plane independent of cluster size. Likewise, the copper clusters are not perturbed greatly, see Table 1. The dimer bond length decreases by only 0.0004 Å upon CH_4_ complexation. In the trimer, the Cu_b_–Cu bonds lengthen by 0.02 Å, and the remote Cu–Cu bond contracts by about 0.04 Å when complexed to methane. In the tetramer, methane complexation leads to the Cu_b_–Cu bonds increasing by 0.03 Å, whereas the remote Cu–Cu bonds decrease by ∼0.04 Å (with the central Cu_b_–Cu bond increasing by 0.02 Å. Only slight modifications of the methane after adsorption are also reflected in the IRMPD spectrum, where the experimental bands closely match the values for free CH_4_, perhaps with a small blue-shift for ν_2_. Notably, the shifts are comparable among the three copper cluster sizes.

**Table 1:** Structural Parameters of the Bare Cu_*n*_^+^ Clusters and of Their Complexes with Methane Calculated at the PBE/def2-TZVPP Level^a^

species	*r*(Cu–Cu) (Å)	*r*(Cu–C) (Å)	*r*(Cu–H_b_) (Å)	*r*(C–H) (Å)
CH_4_				1.096 (4)
Cu_2_^+^	2.401			
Cu_2_^+^(CH_4_)	2.401	2.176	1.884 (2)	1.094 (2), 1.125 (2)
Cu_3_^+^	2.370 (3)			
Cu_3_^+^(CH_4_)	2.329, 2.390 (2)	2.196	1.890 (2)	1.094 (2), 1.123 (2)
Cu_4_^+^	2.418 (4)			
Cu_4_^+^(CH_4_)	2.387, 2.381 (2), 2.447 (2)	2.241	1.922 (2)	1.094 (2), 1.120 (2)

a The number of equivalent bonds are in parentheses.

**Figure 5:**
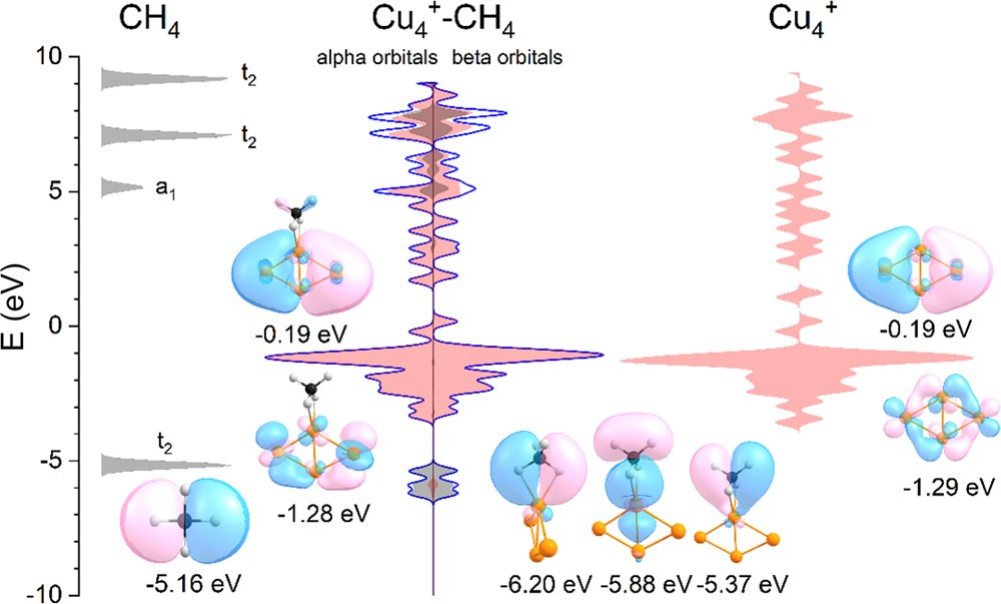
Orbitals for CH_4_ (left, gray shade), Cu_4_^+^ (right, red shade), and Cu_4_^+^–CH_4_, isomer 4A (middle) close to the HOMO–LUMO gap. For the Cu_4_^+^–CH_4_ complex, the projected contributions of CH_4_ and Cu_4_^+^ to the total orbital are calculated and represented in the middle figure with the gray and red shades, respectively. Selected alpha orbitals are graphically represented with their corresponding energy.

A better understanding of the nature of the interaction between Cu_4_^+^ and methane requires an inspection of the orbitals, as shown in Figure 5. Here, one sees a comparison between the orbitals for methane (left), Cu_4_^+^ (right), and the Cu_4_^+^–CH_4_ complex (middle). For the latter, the projected contributions of CH_4_ and Cu_4_^+^ are represented by the gray and red shadings, respectively.^47^ It can be seen that many orbitals are localized on either the copper cluster or methane, for instance those close to the HOMO–LUMO gap. But there are also orbitals with mixed character, in particular, those around −6 eV, which are mostly localized on CH_4_. These orbitals evolve from the *t*_2_ orbitals of CH_4_ that donate electron density to the copper cluster. These orbitals have been stabilized by the interaction with the cluster, that is, have adopted bonding character. Because of the symmetry of these orbitals, the H_b_CH_b_ and H_u_CH_u_ parts of methane are affected similarly by interaction with the copper cluster. Mixing of the *t*_2_ and *a*_1_ antibonding orbitals of CH_4_ also occurs with the Cu_4_^+^ orbitals above +5 eV (as shown by the blue line). In contrast, the Cu_4_^+^ orbitals near +1 eV have been destabilized, without extensive mixing.

**Figure 6:**
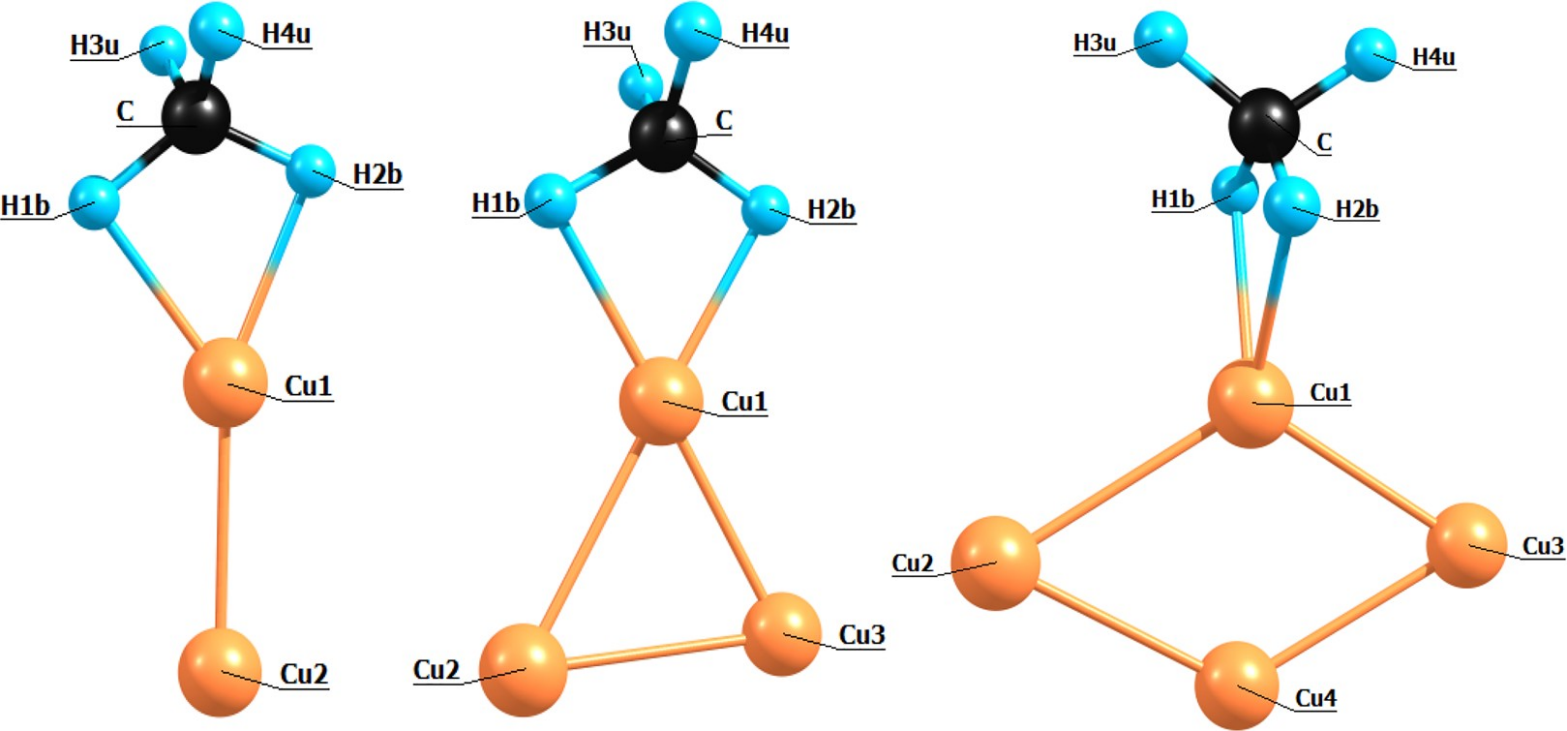
Atomic labeling of structures 2A, 3A, and 4A used to describe Mulliken charges in Table 2.

Further indications for the extent of charge transfer are found in an analysis of the Mulliken atomic charges, listed in Table 2 with atom labels shown in Figure 6, that show a charge decrease for all Cu atoms, but especially for the ones bound to CH_4_, which accept 0.25e, 0.25e, and 0.39e for *n* = 2–4, respectively. In turn, the charge on CH_4_ is net +0.39e for Cu_4_^+^–CH_4_, followed by +0.39e and +0.41e for *n* = 3 and 2, respectively. A dominant negative charge of −0.08 e resides on the carbon (decreasing to −0.09 and −0.11e for *n* = 3 and 2) compared with −0.39 in free CH_4_.

**Table 2:** Mulliken Charges for Structures 2A, 3A, and 4A^a^

	free CH_4_	2A	3A	4A
C	–0.392	–0.088	–0.113	–0.081
H1b	0.098	0.097	0.106	0.097
H2b	0.098	0.097	0.106	0.097
H3u	0.098	0.151	0.144	0.138
H4u	0.098	0.151	0.144	0.138
Cu1		0.252	0.251	0.389
Cu2		0.340	0.181	–0.078
Cu3			0.181	–0.078
Cu4				0.378
net Cu_n_	1	0.592	0.613	0.611
net CH_4_		0.408	0.387	0.389

a Net Cu_*n*_ refers to the charge on the entire copper cluster; net CH_4_ refers to that on methane.

## 4 Conclusion

We have recorded IRMPD spectra for Cu_*n*_^+^–CH_4_ (*n* = 2–4) complexes formed by reacting laser ablation produced Cu_*n*_^+^ clusters with methane in a flow-tube reaction channel in the presence of thermalizing collisions with helium. The spectra for all sizes studied are indicative of physisorption of the methane in η^2^ configurations, with only slight shifts of the methane vibrations with respect to the frequencies for free methane. This is in line with earlier predictions by Maitre and Bauschlicher,^48^ later spectroscopically confirmed by Metz and co-workers,^22^ of the η^2^ configuration for the Cu^+^–CH_4_ originating from donation of electrons from C–H σ (*t*_2_) orbitals to the Cu 4s orbitals. This suggests that the binding between Cu^+^ and CH_4_ has a partially covalent nature.

Our DFT calculations for *n* = 2–4 indicate a slight elongation of the CH_4_ molecule in the planes parallel and perpendicular to the cluster for all cluster sizes. This deformation originates from the charge transfer from the metal cluster to methane in the Cu_*n*_^+^–CH_4_ complex. Transition state calculations for Cu_4_^+^–CH_4_ show that dissociative chemisorption requires crossing a significant barrier, which could not be overcome with current experimental conditions. All in all, it is clear that the CH_4_ binds more tightly to the small clusters: 0.83 < 0.75 < 0.62 eV for *n* = 2–4, respectively. According to Metz and co-workers, the binding energy of the first methane to atomic Cu^+^ is 0.99 eV, which is consistent with the trend seen here.^22^

## Supporting Information Available

Mass spectrum of formed reaction products and comparison between experimental IRMPD spectra with calculated spectra of additional geometric structures (PDF)
