# Supplementary material for: IR Spectroscopic Characterization of Methane Adsorption on Copper Clusters Cun+ (n = 2–4)
Source: J Am Soc Mass Spectrom. 2022 Apr 12;33(8):1393–400. doi: 10.1021/jasms.2c00046 (PMC9354255; doi:10.1021/jasms.2c00046)
Supplement: Supplementary file 1 — js2c00046_si_001.pdf [file js2c00046_si_001.pdf]

SUPPORTING INFORMATION

for

IR spectroscopic characterization of methane adsorption on copper clusters Cu_n^+ (n=2-4)

Olga V. Lushchikova,[§, +] Stijn Reijmer,[§] P.B. Armentrout,[†] and Joost M. Bakker[§,*]

[§]*Radboud University, Institute for Molecules and Materials, FELIX Laboratory, Toernooiveld 7, 6525 ED Nijmegen, The Netherlands*

[†]*Department of Chemistry, University of Utah, 315 S. 1400 E. Room 2020, Salt Lake City, Utah 84112, United States*

+Institut für Ionenphysik und Angewandte Physik, Universität Innsbruck, Technikerstraße 25, 6020 Innsbruck, Austria

**corresponding author: joost.bakker@ru.nl*

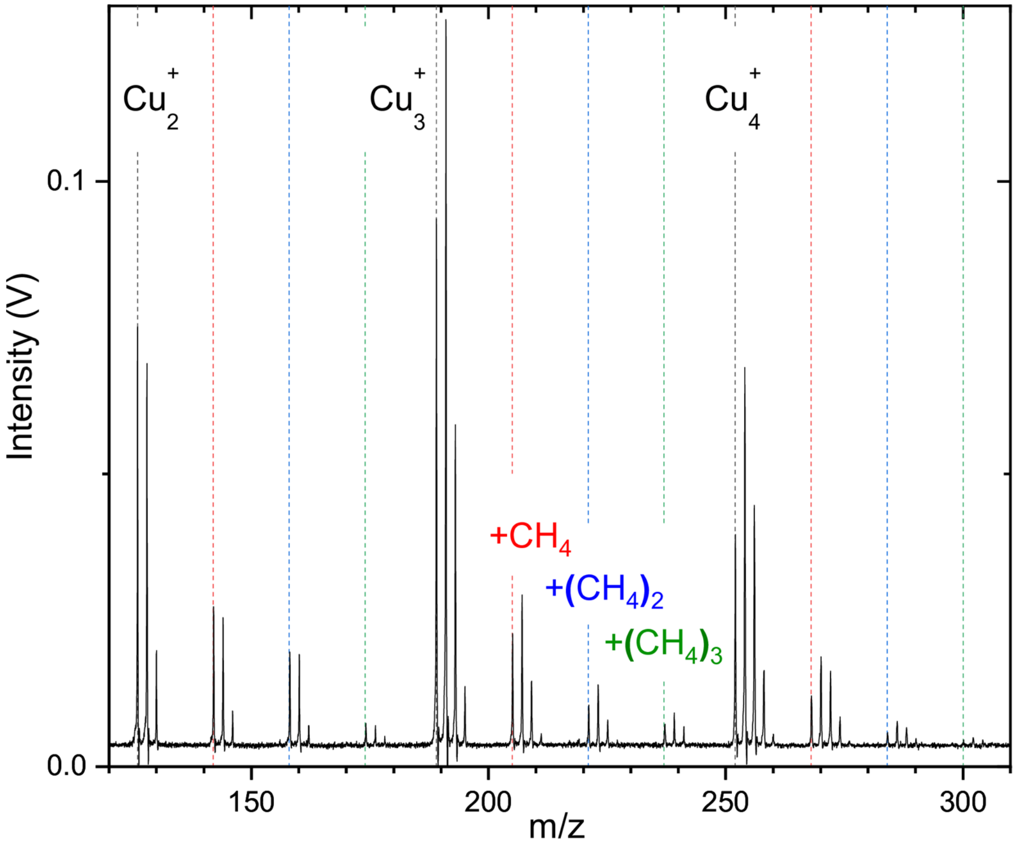

Figure S1: Mass spectrum of the products formed in the reaction between Cu_n^+ (n=2-4) clusters with methane. The masses of bare ^{63}Cu clusters, clusters with one, two, and three methane molecules are indicated with black, red, blue, and green dashed lines, respectively. Natural abundances: ^{63}Cu, 69%, ^{65}Cu, 31%.

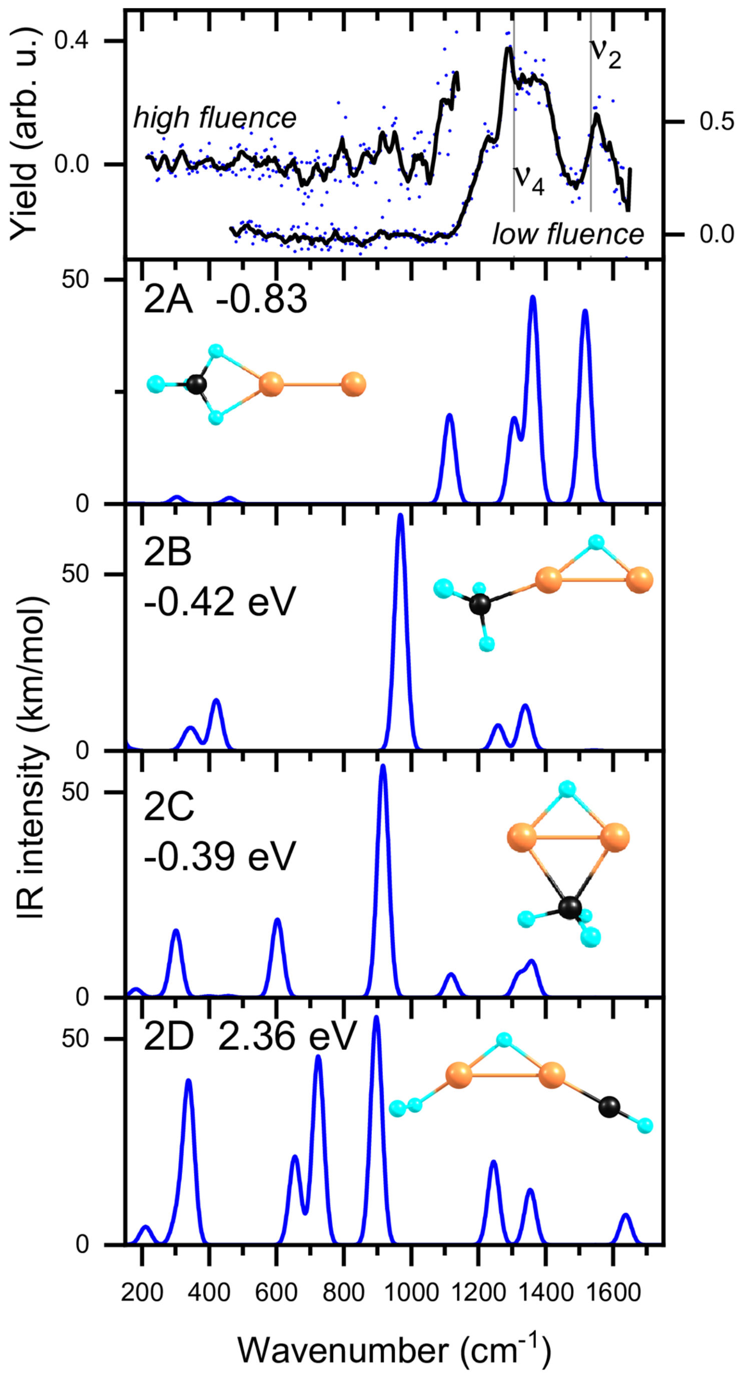

Figure S2: Experimental IRMPD spectrum of Cu_2^+-CH_4 (top panel) recorded under low and high-fluence conditions. The raw experimental data (blue scatter) are accompanied by a three-point adjacent average (black line). The gray vertical lines indicate the observed vibrational frequencies of free CH_4. Lower panels (blue trace) show calculated IR spectra of possible product structures. Each structure is accompanied by the energy with respect to the Cu_2^+ and CH_4 reactants. Cu, C, and H atoms are represented by orange, black, and cyan spheres, respectively.

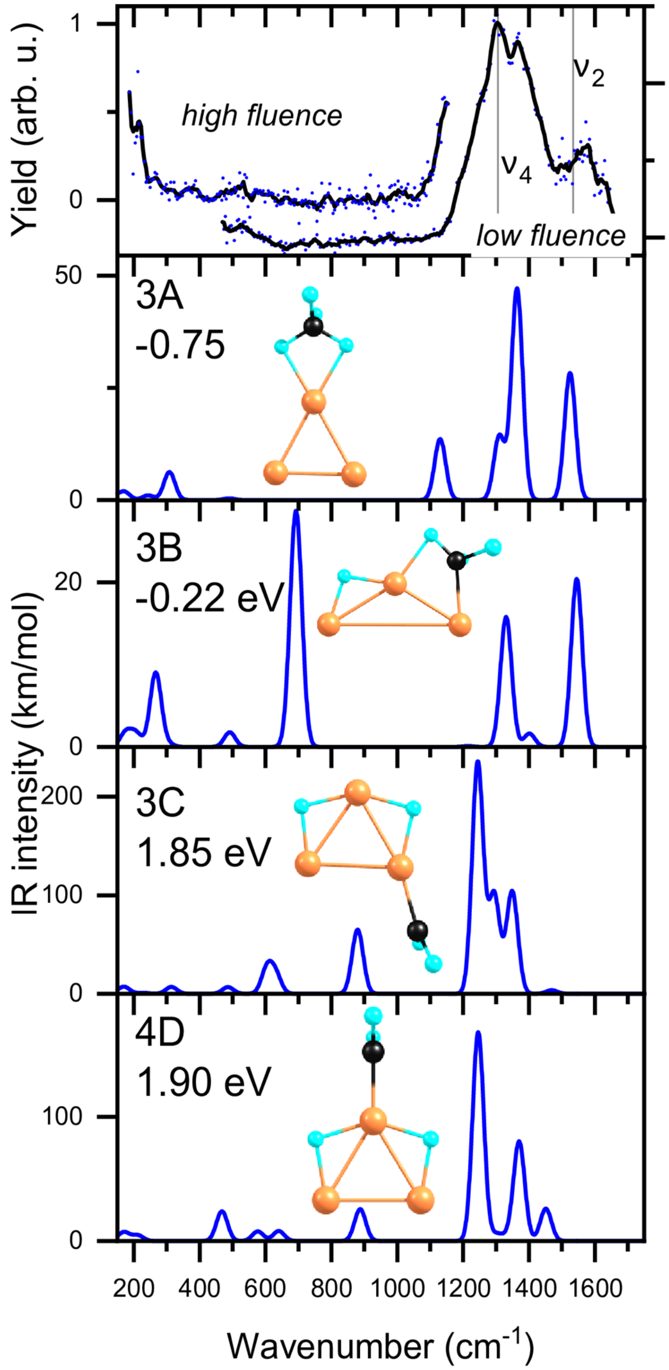

Figure S3 Top panels: experimental IRMPD spectrum of Cu_3^+-CH_4; lower panels: calculated IR spectra of possible product structures. For further details, see caption Figure S2.

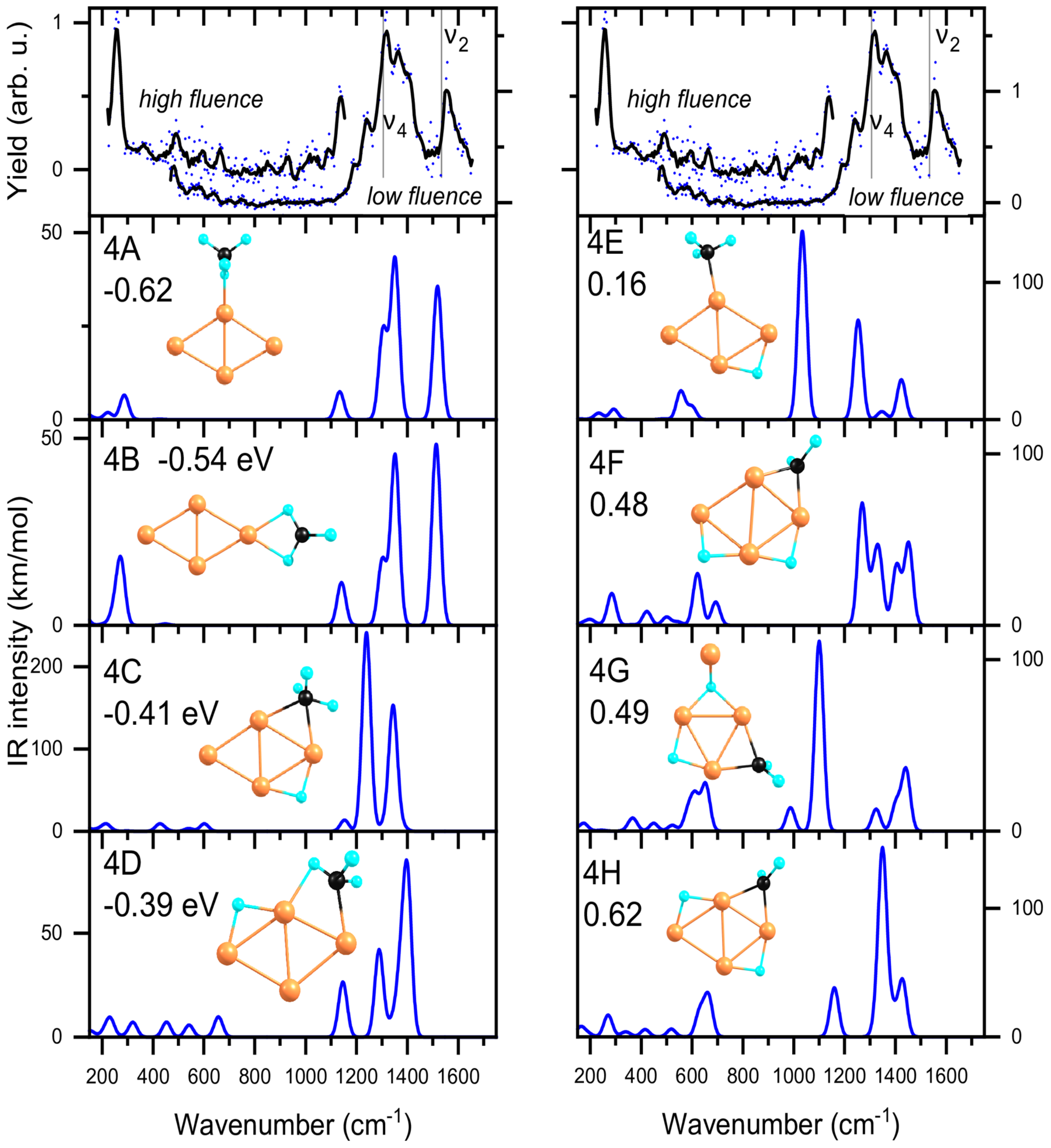

Figure S4 top panels: experimental IRMPD spectrum of Cu_4^+-CH_4; lower panels: calculated IR spectra of possible product structures. For further details, see caption Figure S2.
